# Which Expectations to Follow: The Impact of First- and Second-Order Beliefs on Strategy Choices in a Stag Hunt Game

**DOI:** 10.3390/bs13030228

**Published:** 2023-03-06

**Authors:** Thomas Neumann, Paul Bengart, Bodo Vogt

**Affiliations:** 1 Health Services Research, University of Siegen, 57076 Siegen, Germany; 2 Empirical Economics, Otto von Guericke University Magdeburg, 39106 Magdeburg, Germany; 3 University Department of Neurology, Otto von Guericke University Magdeburg, 39120 Magdeburg, Germany; 4 Research Campus STIMULATE, Otto von Guericke University Magdeburg, 39106 Magdeburg, Germany; 5 Institute of Social Medicine and Health Systems Research, Otto von Guericke University Magdeburg, 39120 Magdeburg, Germany; 6 Center for Behavioral Brain Sciences (CBBS), 39106 Magdeburg, Germany

**Keywords:** coordination games, first-order beliefs, second-order beliefs, risk attitudes, uncertainty, experimental economics

## Abstract

Many situations require coordinated actions of individuals to achieve common goals. Such situations include organizing mass protests or adjusting behavior to new behavioral recommendations that aim to slow down the spread of a contagious disease. However, there is a risk of coordination failure in such situations that can lead to a worse outcome for those who acted in a coordinated manner than for those who chose not to. In this paper, we investigate the main determinant of individuals’ decisions in these situations to determine whether beliefs regarding the action of others (empirical expectations), beliefs regarding others’ beliefs (normative expectations), or risk attitudes are dominant determinants. To this end, we conducted an experiment analyzing the relationship between an individual’s choices in a stag hunt game, their probabilistic empirical and normative expectations (i.e., first-order and second-order beliefs, respectively), and their risk attitudes. Our central finding is that expectations, not risk attitudes, explain individuals’ strategy selection. In addition, we found evidence that normative expectations are a better predictor of strategy selection than empirical expectations. This could have implications for developing more targeted strategies intended to promote new behavioral standards and to guide individuals’ behavior toward a welfare-maximizing equilibrium.

## 1. Introduction

Coordinating one’s own actions with those of others can help in achieving goals that benefit all involved and that an individual alone cannot otherwise achieve. Given the uncertainty about others’ actions and multiple equilibria, however, there is a risk of coordination failure, which can hinder the achievement of the best possible outcome. A frequently used example of a real-world situation in which the just described uncertainty applies is one of coordinating (mass) protests [1,2,3,4]. The success of a protest is strongly related to the number of protest participants. Joining a large-scale protest can be a powerful strategy to trigger a political change or even collapse a political system, as happened following the German Democratic Republic protests in 1989 and the Arab Spring of 2010 onward. In contrast, joining a protest in which only a few people participate is far less effective in achieving protest goals, and it has a higher risk of sanctions, such as imprisonment or fines. Such participation would put the protester in a worse position than staying away from the protest. In these situations, uncertainty about others’ actions arises because communication with all other potential protestors is hardly feasible, and promises made are not binding.

Another example of a situation in which individuals are better off when they coordinate their actions is one in which individuals have to decide whether or not to adhere to measures intended to limit the spread of contagious diseases (e.g., COVID-19). Such measures can include (voluntarily) wearing masks in stores, which is effective only if a majority of the population chooses to wear a mask. Although wearing a mask can reduce the risk to oneself and others of contracting a contagious disease, individuals can derive disutility from mask-wearing, which they might perceive as uncomfortable and can carry financial costs arising from mask purchases. However, not wearing a mask, if everybody else does, is not only associated with a higher risk of becoming infected, it can also have negative social consequences of violating the social norms, such as being avoided by others or being perceived as antisocial [5]. However, such negative social consequences are less likely to occur when hardly anyone wears a mask. One possible coordination equilibrium could be that everyone wears a mask (i.e., mask-wearing becomes a social norm) and another that nobody does (i.e., mask-wearing does not become a social norm). Therefore, when facing a coordination problem, relying on social norms can be helpful to individuals in achieving coordinated actions. According to Krupka et al. [6], social norms can be defined as “jointly recognized beliefs, among members of a population, regarding the appropriateness of different behaviors” (p. 4). As Krupka and Weber [7] suggest, concerns about violating social norms lead individuals to choose the strategy they perceive to comply with social norms. Well-established social norms can therefore serve as a focal point for coordination.

Beliefs or expectations that lead people to behave in accordance with what they perceive as a social norm can be divided into two dimensions: empirical expectations and normative expectations. According to Bicchieri [8], empirical expectations are beliefs about others’ actions, and normative expectations are beliefs about others’ beliefs, whereby the former might depend on the latter [9,10,11]. The empirical dimension of expectations is also referred to as first-order beliefs (FOBs), and the normative dimension as second-order beliefs (SOBs).

Although there is some evidence to indicate that FOBs and SOBs could play an important role in their decision-making process under social uncertainty [12,13,14,15], only limited research has been conducted that systematically investigates their impact on coordinated behavior, which is especially true for SOBs. Therefore, the main purpose of the present study was to gain a further understanding of how and to what extent FOBs and SOBs influence individuals’ behavior in coordination situations. Knowing what factors are primarily responsible for the success or failure of coordination could have implications for developing more targeted strategies intended to guide individual behavior toward a welfare-maximizing equilibrium.

Coordination situations such as those described in the examples at the beginning of the introduction can be naturally modeled as a game under several sources of uncertainty, in which the individuals’ payoffs depend on their own actions, others’ actions, and some unknown economic fundamentals [16]. The classical problem individuals in such situations have to solve relates to the tradeoff between uncertainty reduction and the resulting outcome [17]. Symmetric games with multiple equilibria, such as coordination games, serve as typical examples of such a tradeoff situation involving uncertainty.

As the literature from other fields of research indicates (e.g., moral behavior of athletes), SOBs might be a better predictor of behavior under social uncertainty than FOBs [18,19]. Despite the possibility that the SOBs could outperform the FOBs in predicting individual behavior in coordination situations, there is only limited research that has directly examined the effect of SOBs on participants’ behavior in coordination games. Moreover, most of the few existing studies on SOBs measured this variable only as a qualitative value, e.g., [9,20]. Data on qualitative SOBs, however, do not allow for any conclusions about the degree of confidence surrounding individuals’ statements about their SOBs. One way of obtaining this information is to elicit the SOB, as well as the FOB, as a probabilistic value [21]. Of course, individual decisions are not solely driven by belief, but also by other factors, such as risk attitudes [16,17,22]. For example, depending on their risk attitudes, individuals might derive disutility from risk, which can lead them to choose an action that minimizes the risk of obtaining the poorest outcome rather than an action that maximizes the outcome. In light of the above, the question arises as to which of the person-related factors—empirical expectations (i.e., FOBs), normative social expectations (i.e., SOBs), and risk attitudes—is the main determinant of the behavior in situations in which coordinated actions lead to beneficial outcomes?

One of the most widely used types of coordination games is the stag hunt game, which we have used as the basis for this study, aiming to provide an answer to the above-mentioned question. Although many researchers consider it a coordination game [3,4,23], the story behind the stag hunt game has elements of cooperation. This game creates a virtual situation in which two hunters decide whether to cooperate and jointly hunt a stag or not to cooperate and individually hunt a rabbit [24,25]. In other words, they decide whether to select a safe (risk-free) strategy (“rabbit”) or an uncertain strategy (“stag”). In contrast to this structure, we used a stag hunt game version in which both strategies provided uncertainty regarding outcomes. In the game this study uses, the Rabbit strategy (Strategy B) provides a security level, which is defined as “the minimal payoff a player can secure himself by choosing Rabbit” [26] (p. 2052) regardless of the other player’s actions. However, the player still has a chance to obtain a higher (but not maximal) payoff if the two players coordinate on the risk-dominant equilibrium (Strategy B, Strategy B). As is usual, the Stag strategy (Strategy A) provides the maximum payoff if the two players coordinate on the Pareto-efficient, payoff-dominant equilibrium (Strategy A, Strategy A). In addition to the stag hunt game, we used different tasks to elicit participants’ risk attitudes and their qualitative and probabilistic FOBs and SOBs.

Our data indicated that the individual’s stated beliefs largely explain their behavior, with SOBs explaining more variance in strategy choices than FOBs. However, our results do not provide evidence of a direct relationship between individuals’ risk attitudes and their behavior. Yet, we did find a small but significant effect of risk attitudes on both FOBs and SOBs, suggesting we cannot rule out an indirect effect of risk attitudes on the participants’ strategy choices. Based on these findings, we argue that in analyzing the behavior in stag hunt games, individual SOBs and FOBs are more important than their risk attitudes.

The paper is structured as follows. In Section 2, we provide information about the game design and main research hypotheses. Section 3 explains the experimental procedure, followed in Section 4 by a presentation and discussion of the results. Finally, Section 5 concludes the discussion.

## 2. Game Design and Hypotheses

**Figure 1:**
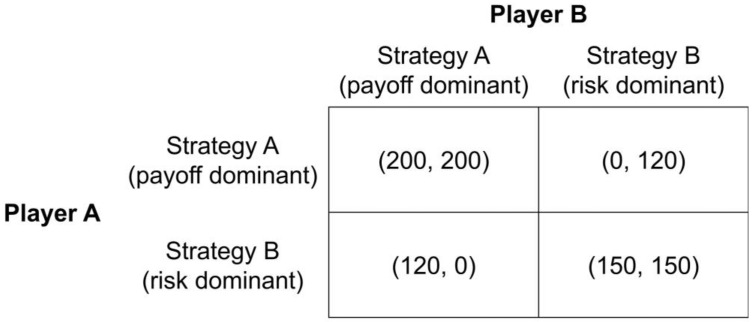
Payoff structure of the stag hunt game.

Our experiment is based on a two-person stag hunt game in which two players were asked to choose between two possible strategies, Strategy A or Strategy B. The return on the selected strategy depends on each player’s own decision and on that of an additional player. Figure 1 shows the payoff structure of the game used in our experiment.

### 2.1. Equilibrium Selection in a Coordination Game

As can be seen in Figure 1, the game has two equilibria. If the row player (column player) chooses Strategy A, the best response of the column player (row player) is also to choose Strategy A. In contrast, if one player chooses Strategy B, the other player’s best response would be to choose Strategy B. Individuals who strive to maximize the individual payoff and those who strive to maximize the joint monetary outcome would prefer the Pareto-efficient Nash equilibrium (Strategy A, Strategy A). If, however, risk attitudes are the driving force behind the strategy selection, the Pareto-inefficient Nash equilibrium (Strategy B, Strategy B) could also become a stable equilibrium.

Following the two selection criteria introduced by Harsanyi and Selten [27], the Nash equilibria (Strategy A, Strategy A) is payoff dominant, and (Strategy B, Strategy B) is risk dominant. In addition, this game includes one mixed strategy Nash equilibrium, where each player chooses Strategy A with a 0.65 probability.

### 2.2. Belief Elicitation

As with many other studies [13,28,29,30] we elicited the players’ FOB directly by asking them to explicitly predict their partner’s strategy choice (qualitative FOB). Additionally, we asked them to indicate, using a number between 0 (not confident at all) and 100 (fully confident), the extent to which they were confident that their prediction was accurate (*probabilistic FOB*). These numbers were divided by 100 to calculate the probabilities. The players were rewarded according to a *quadratic scoring rule* (QSR) adopted from Nyarko and Schotter [28] and Berninghaus et al. [13], which is “by far the most popular proper scoring rule” [31] (p. 241) and which is based on the axiomatic characterization formulated by Selten [32]. An incentivized elicitation of beliefs was thought to be necessary according to Gächter and Renner [33] and Trautmann and van de Kuilen [34], who demonstrated that the accuracy of stated FOB increases when players are rewarded. Moreover, Palfrey and Wang [35] found that the properness of the reward function is relevant in terms of truthful belief revelation. A reward function (or scoring rule) is defined as proper “if it gives a risk-neutral decision maker an incentive to report truthfully” [36] (p. 105). Several studies, such as Croson [37], Nyarko and Schotter [28], Costa-Gomes and Weizsäcker [38], Gächter and Renner [33], and Neumann and Vogt [39] have examined the question of whether belief elicitation affects behavior in games. The evidence these studies provide is mixed. Schlag et al. [40] concluded that, given the relatively limited number of studies that have assessed the effect of belief elicitation on behavior, “we need to improve our understanding about the interactions between belief elicitation and game play” (p. 485). However, Hoffmann [41] interpreted the literature as indicating that eliciting beliefs does not always affect game play.

The (proper) QSR we used is designed to be optimal for a risk-neutral player to report their true belief [42]. Despite its complexity compared to simpler belief elicitation mechanisms, such as the frequency [43] and the interval method [44] its accuracy in eliciting beliefs does not suffer in comparison to the simpler mechanisms [31].

In our experiment, we used functions of the following types:

– If the player’s partner chooses the predicted strategy, the payoff is:

$$100\cdot \left[1-{\left(1-\frac{p}{100}\right)}^{2}\right]\;\left\{in\;Points\right\}$$

– If the player’s partner does not choose the predicted strategy, the payoff is:

$$100\cdot \left[1-{\left(\frac{p}{100}\right)}^{2}\right]\;\left\{in\;Points\right\}$$

We used a procedure similar to that described above to elicit the participants’ SOBs. First, we asked participants to indicate their beliefs regarding their partner’s beliefs about which strategy they had chosen (*qualitative SOB*). Second, we asked participants to indicate their confidence level (0–100) in answering the former question (*probabilistic SOB*). Again, we used the QSR as an incentive mechanism to encourage participants to reveal their true beliefs.

### 2.3. Lottery Choices

We used a lottery choice task, which we adapted from Holt and Laury [45] to elicit the players’ risk attitudes. In this task, we asked participants to compare lotteries that had the same payoff structure as that of the stag hunt game (see Figure 1). Thus, for example, the possible payoffs from choosing Lottery A coincided with the possible payoffs of choosing the risky choice (Strategy A) in the coordination game. In this task, we varied possible probabilities across lotteries “simulating” different decisions in which the player knew the probability with which the other player would decide to select Strategy A. The lotteries we used are presented in Table 1.

According to studies on risk preferences, such a multiple-price list design can help to identify degrees of risk aversion [45]. A risk-neutral individual, for example, would switch from Lottery A to Lottery B between no. 5 and 6 since, after no. 5, Lottery B’s expected payoff becomes greater than that of Lottery A. The further down the switching point, the higher the degree of an individual’s risk aversion, and vice versa.

### 2.4. Research Hypotheses

We translated our research question into three testable hypotheses. Given the literature suggesting a relationship between individuals’ FOB and their behavior in interpersonal situations [13,28,38], we formed the first hypothesis:

**H1a:**  *The players* *’ empirical expectations (i.e., FOBs) determine their strategy selection in the 2 × 2 coordination game.*

Because individuals’ first-order beliefs can strongly depend on their second-order beliefs [9,10], we expected that:

**H1b:**  *The players* *’ normative social expectations (i.e., SOBs) determine their strategy selection in the 2 × 2 coordination game.*

Heinemann et al. [16] demonstrated that individuals’ risk attitudes (elicited by lottery choices) affected their behavior in coordination games. They showed that risk-averse players avoid strategic uncertainty. Schmidt et al. [17] reported similar results. Furthermore, Guarin and Babin [22] found that risk attitudes have a statistically significant effect on players’ strategy choices in a stag hunt game, though the effect was small, as the authors noted. We, therefore, expected that players’ risk attitudes would affect their behavior in the coordination game. Specifically, we expected risk-averse players to be more likely to choose Strategy B and, thereby, compared to those who are risk-seeking, to reduce strategic uncertainty.

**H2:**  *The players* *’ risk attitudes determine their strategy selection in a 2 × 2 coordination game.*

Related to H1a and H1b, we also analyzed whether individuals best respond to their stated probabilistic FOB and SOB. Here, we refer to Rey-Biel [30], who defined “best replying behavior as choosing the action that gives the highest expected payoff given the distribution of beliefs stated” (p. 581). The literature provides various examples of studies that have demonstrated that the majority of players choose the best response strategy [28,30]. However, several studies also show that players often fail to respond best to their stated beliefs [16,29,38].

## 3. The Experiment

To answer the research question and test our hypotheses, we designed an experiment consisting of four tasks. In this section, we provide an overview of the design and procedure of each task. We performed the experiment in the MaXLab, the experimental laboratory at the University of Magdeburg. Participants were recruited using hroot [46] from a pool primarily consisting of students from various faculties. We programmed and ran the experiment using the z-Tree software [47]. All instructions were given in German (the English translation can be found in Appendix A). The study was conducted in accordance with ethical standards for human research and under the terms and conditions of the MaXLab.

In the first stage of our experiment, two participants were randomly matched to play a (one -shot) stag hunt game (first task), as explained in Section 2.1. Participants were randomly assigned to play the game as Player A or Player B, and were then asked simultaneously to choose one of the two possible strategies: Strategy A or Strategy B. After making their strategic decision; the players were asked to complete the belief elicitation tasks assessing participants’ FOBs (second task) and SOBs (third task) (see Section 2.2). To assess and reduce potential task-order effects, we varied the order of the first to third task (see Table 2).

In the second stage of the experiment, we used the lottery choice task (fourth task) to identify each player’s risk attitude. The participants were shown a table that contained two lottery tickets, Lottery A [G_1A_, p%; G_2A,_ (100 − p)%] and Lottery B [G_1B_, p%; G_2B,_ (100 − p)%], as explained in Section 2.3. For each of the thirteen pairs of lotteries, they were asked to specify which lottery they preferred, with the option to indicate “indifferent”. Contrary to the representation in Section 2.2, we changed the order of the probabilities (p) and the payoffs (G) in the lottery tickets. Thus, the participants were shown the payoffs first to enhance their understanding of the concept.

All payoffs were given in points. At the very end of the experiment, these points were converted into euros at a previously known exchange rate of €6 per 100 points. Table 2 summarizes the possible payoffs that participants could achieve in each experimental task and provides an overview of the task order across the experimental treatments.

At the end of the experiment, participants received their payoffs separately and sequentially. For the first stage of the experiment, participants were paid according to the points they obtained in the coordination game (first task), the FOB elicitation task (second task), and the SOB elicitation task (third task). To give an example: a player who chose Strategy B while their partner chose Strategy A would receive €7.20 (120 points/100 × €6) from the first task. Furthermore, in the second task, this player expected their partner to choose Strategy B with a confidence of 70% (i.e., p = 70). Since this guess is not correct, their payoff from this task would be calculated using the second equation in Section 2.2: 100 × [1 − (70/100)^2^] = 51 points (or €3.06). In the third task, the same player expected with 80% (i.e., p = 80) that their partner expected them to choose Strategy A. Their partner indeed expected the player to choose Strategy A so that the player’s guess is correct, and the first equation from Section 2.2 is applied for payoff calculation: 100 × [1 − (1 − 80/100)^2^] = 96 points (or €5.76). Thus, the player from the example would receive a total payoff of €16.02 in the first stage of the experiment.

For the fourth task of the experiment (second stage), only one of the lottery choices was realized. For each participant, this decision was determined by drawing one ball from a bingo cage that contained thirteen balls numbered 1 to 13. According to the participants’ choices, they played the preferred lottery (Lottery A or Lottery B) by drawing a ball from a bingo cage containing a specified number of red and blue balls, reflecting the probabilities of the lottery: the number of red balls equated to the probability of payoff one (G_1_) and the number of blue balls equated to the probability of payoff two (G_2_). In the case of indifference, we determined the lottery to be played by the toss of a coin.

Participants were not permitted to communicate with each other at any point during the experiment. They were not provided with any information about their payoffs or the behavior of their partners. In total, 151 subjects participated in the experiment (39 in the first task order condition, 38 in the second task order condition, 36 in the third task order condition, and 38 in the fourth task order condition). However, for the analyses, we excluded 9 participants whose risk attitudes we could not determine because, in the lottery choice task, they switched between the lotteries more than once. This left a final sample size of 142 (38 in the first treatment, 34 in the second treatment, 34 in the third treatment, and 36 in the fourth treatment).

## 4. Results

In this section, we first provide a descriptive analysis of participants’ responses. Next, we analyze the effects of participants’ probabilistic FOBs, probabilistic SOBs, and risk attitudes on their strategy choices using a series of regressions to answer our hypotheses. Subsequently, we test whether risk attitudes impacted probabilistic FOBs and probabilistic SOBs, and we assess whether the task order affected participant responses.

### 4.1. Descriptive Analysis

As explained in previous sections, we presented the coordination game and lottery choices in similar kinds of setup. For the stag hunt game, we asked participants to choose between an option with an uncertain payoff (Strategy A) and an alternative with a less uncertain payoff (Strategy B). Our data show that the majority (83.10%) of the players chose Strategy A in the coordination game (see Table 3). Also, the majority of the players stated they believed the other player chose Strategy A and believed the other player believed they themselves had chosen Strategy A.

**Figure 2:**
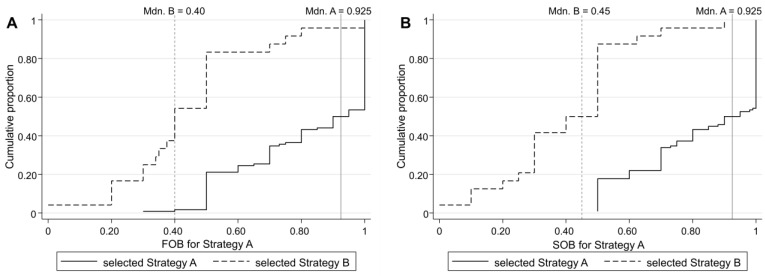
CDFs of (**A**) probabilistic FOB and (**B**) probabilistic SOB of participants who selected Strategy A and of those who selected Strategy B. Mdn. A = Median (**A**) FOB and (**B**) SOB of those who selected Strategy A; Mdn. B = Median (**A**) FOB and (**B**) SOB of those who selected Strategy B.

The next figure shows the cumulative distribution functions (CDFs) of the probabilistic FOB regarding the likelihood that the other player chose Strategy A (Figure 2A) and the cumulative distributions of the probabilistic SOBs regarding the likelihood that the other player believes they themselves had chosen Strategy A (Figure 2B). Note that we transformed the stated beliefs ranging from 0 to 100 into the unit interval of [0,1]. 

By comparing Figure 2A to Figure 2B, we could observe that the CDFs and the medians of probabilistic FOBs and SOBs were quite similar. However, comparing the CDFs of those who chose Strategy A to the CDFs of those who chose Strategy B, we could see that the CDF for the former dominates the CDF for the latter, which applies equally to FOBs and SOBs. Consequently, the median of probabilistic FOBs and SOBs for those who chose Strategy A (0.9 and 0.9, respectively) is larger than for those who chose Strategy B (0.4 and 0.4, respectively).

**Figure 3:**
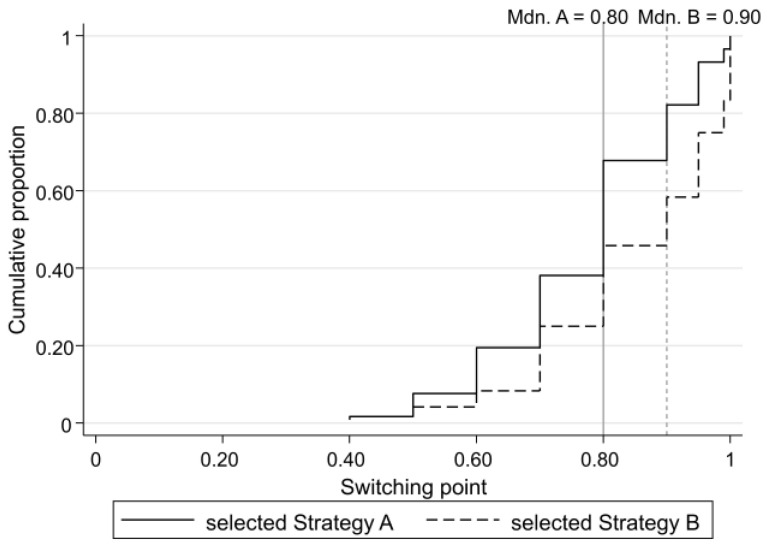
CDFs and the medians of switching points of participants who selected Strategy A and of those who selected Strategy B.

The following figure (Figure 3), which is plotted in the same manner as Figure 2, displays the CDFs of the switching points (defined as the lowest probability of receiving the high payoff in Lottery A) in the case (No. of pairs) that the participant preferred Lottery A to Lottery B for the first time. Always choosing Lottery B is reflected by a switching point of 1, as we assume that players would opt for Lottery A if the probability of receiving the high payoff is equal to 1 in the lottery choice task.

As Figure 3 shows, there are only slight differences between the CDFs and the medians of the switching points between a Strategy A player and a Strategy B player, i.e., the two player types had similar risk attitudes. Comparing Figure 2 to Figure 3 indicates that the participants’ strategy choices were determined more by their beliefs than by their risk attitudes.

### 4.2. Determinants of the Players’ Strategy Choices

We estimated logit regressions to gain a deeper understanding of how risk attitudes and probabilistic FOBs and SOBs affect behavior in the coordination game. Prior to conducting logit regression analyses, we first performed a correlation analysis and a Farrar-Glauber test [48] to assess whether there is multicollinearity between the predictors since comparing Figure 2A to Figure 2B led us to the suspicion that the predictors FOB and SOB might be highly correlated. The results suggest that indeed the probabilistic FOBs and SOBs are highly correlated (*r*(149) = 0.808, *p* < 0.001). Likewise, the Farrar-Glauber test results reject the hypothesis of no multicollinearity between FOBs and SOBs (*F*(2, 149) = 280.42, *p* = 0.004). Regarding the switching points, the correlation analysis revealed only a weak negative relationship between the switching points and the two belief types FOB (*r*(142) = −0.203, *p* = 0.016) and SOB (*r*(142) = −0.205, *p* = 0.014). According to the Farrar-Glauber test, using the switching points together with the FOB (*F*(2, 140) = 6.00, *p =* 0.153) in one model and switching points with the SOB (*F*(2, 140) = 6.17, *p =* 0.149) in another model would not create a multicollinearity problem. 

The results from the multicollinearity tests described above led us to fit two separate logit regression models. In both models, the dependent variable was the observed strategy selection (0 = Strategy A, 1 = Strategy B). Model 1a included the participants’ probabilistic FOB [0,1] (that the partner had chosen Strategy A) and their risk attitudes (i.e., switching points) [0,1]. Model 1b included participants’ probabilistic SOB [0,1] (that the partner believes they themselves had chosen Strategy A) and their risk attitudes. Table 4 shows the results of the logit regression analyses.

The results drawn from Model 1a indicate that the stated probabilistic FOB had a significant (*p* < 0.001) negative impact on the selection of Strategy B. This means, the more confident participants were that the other player would choose the payoff-dominant Strategy A, the less likely they were to choose the risk-dominant Strategy B. Further, we noted a positive effect of risk attitudes on the selection of Strategy B, which, however, was not significant (*p* = 0.309). From Model 1b results, we can see that the probabilistic SOB had a significant (*p* < 0.001) negative effect on the selection of Strategy B, while risk attitude did not (*p* = 0.234).

Comparing McFadden’s pseudo R^2^s of the models suggests that Model 1b explains more of the variance in strategy selection than Model 1a does. A subsequent bootstrap analysis with 1000 replications revealed that the standard error interval [0.279, 0.483] of pseudo *R*^2^ of model 1a does not overlap with the pseudo *R*^2^ of Model 1b. Next, to assess the extent to which each of the variables contributed to the overall fit of model 1a and Model 1b, we performed a dominance analysis. According to the results, FOB contributed 93.52% and risk attitudes only 6.48% to McFadden’s *R*^2^ of 0.381 of Model 1a. In Model 1b, SOBs contributed 94.77% and risk attitudes 4.10% to McFadden’s *R*^2^ of 0.505. Based on these results, we concluded that the impact of risk attitudes on strategy choices is negligible.

Our data, therefore, support our hypotheses that players’ first-order beliefs (H1a) and their second-order beliefs (H1b) determine their strategy selection; however, the data do not support the hypothesis (H2) that players’ risk attitudes determine their strategy selection.

In the next regressions, we tested whether risk attitudes influenced the participants’ beliefs. Model 2a has the probabilistic FOB, and model 2b has the probabilistic SOB as the dependent variable. Both models used risk attitudes as the explanatory variable. Table 5 presents the results.

The results of the regression analyses indicate a statistically significant effect of risk attitudes on both the FOB and SOB, although the effect size is small (*R*^2^ = 0.041 and *R*^2^ = 0.042, respectively). The negative coefficient implies that the more risk -seeking the participants were, the lower their belief that their partner had chosen Strategy A, and the lower their belief that their partner believes they themselves had chosen Strategy A.

### 4.3. Best Response Behavior, Risk Attitudes, and Task-Order Effects

In light of these results, the following analysis focuses on determining whether the participants chose the best response to their stated probabilistic first- and second-order beliefs. We observed that 76.76% and 80.28% of the participants best responded to their probabilistic FOB and SOB, respectively. The FOB-based best response rate was higher than the rates Costa-Gomes and Weizsäcker [38] and Rey-Biel [30] reported. In the second step, we performed a binomial test. We found that players responded to their FOBs and SOBs significantly more frequently than if they had chosen their strategy randomly (exact binomial test, two-sided; both with *p* < 0.001, *n* = 142).

In the next analysis, we tested whether the order in which participants completed the tasks affected the responses given in the first task of the experiment. For this purpose, we modified model 1a and model 1b by including treatment as a further predictor variable. In the next step, we calculated the predictive margins of FOBs and SOBs by treatment. A pairwise comparison of the predictive margins of FOB indicated a significant difference only between the second and the fourth treatments (*χ*^2^ = 4.13, *p* = 0.042). The effect of FOBs on strategy choices was 11 percentage points higher in the fourth treatment (order: SOB, FOB, strategy selection, lottery choices) compared to the second treatment (order: strategy selection, SOB, FOB, lottery choices). 

## 5. Discussion

In this study, we investigated whether individuals’ beliefs concerning the others’ behavior (i.e., first-order beliefs (FOBs) or empirical expectations), their beliefs concerning the others’ beliefs (i.e., second-order beliefs (SOBs) or normative expectations), or their risk attitudes determine their strategy selections in a stag hunt game, which is a coordination game variant. To answer this question, we conducted a laboratory experiment in which participants completed four incentivized tasks, which were presented in four different orders to account for possible task-order effects. In the stag hunt game, participants chose between two possible actions, the payoff-dominant Strategy A and the risk-dominant Strategy B. In contrast to many other studies based on the stag hunt game [12,49,50], we used a game version in which both strategies were associated with uncertainty about the payoffs. In the belief elicitation tasks, we collected data on the participants’ qualitative FOBs and SOBs and, in addition, data on the subjective probabilities that participants assigned to the other player’s action (probabilistic FOBs) and the other player’s belief about one’s own action (probabilistic SOBs). The lottery choice task we used to elicit participants’ risk attitudes differed from the strategy choice task because it provided participants with objective probabilities of obtaining different payoffs. The possible payoffs of Lottery A and Lottery B, however, were the same as those of Strategy A and Strategy B in the stag hunt game.

The main finding of this study is that both the probabilistic FOBs and probabilistic SOBs are strong predictors of participants’ strategy choices, thus supporting the hypotheses that FOBs (Hypothesis 1a) and SOBs (Hypothesis 1b), respectively, determine the strategy selection in the 2 × 2 coordination game. According to the results from the dominance analysis, the relative importance of the probabilistic FOBs was about 93% (model 1a), and that of the probabilistic SOBs was about 94% (model 1b). Our data also show that the model with the probabilistic SOBs as the predictor showed a better fit than the one with the probabilistic FOBs. The finding that the majority of the participants in our experiment best responded to their stated probabilistic FOBs and SOBs further supports the dominant role of beliefs in decision-making in coordination situations. Our findings regarding the relationship between FOB and the behavior in the coordination game are thus consistent with those of Büyükboyacı [12] and Berninghaus et al. [13]. Regarding the SOB, there are only a few studies with which to compare our results. One of these is the study of Manski and Neri [21], which elicited probabilistic FOBs and SOBs in a hide -and -seek game. The authors found that the participants’ choices in the game are more consistent with their FOB than with their SOB. However, unlike the stag hunt game, the hide -and -seek game represents a competitive situation with a zero-sum payoff structure. Another study, conducted by Ibanez and Saadaoui [51], found that SOBs had no direct but an indirect effect on strategy selection in a joy-of-destruction game due to their strong influence on the FOBs. From this, we conclude that the role of FOB and SOB depends on the context in which decisions are made.

Another result of this study is that we found no statistically significant evidence that the participants’ risk attitudes directly influenced their strategic decisions in the stag hunt game, leading us to reject Hypothesis 2, stating that risk attitudes determine the strategy selection in a 2 × 2 coordination game. This is also evident from the results of the dominance analysis, which show that risk attitudes were of low relative importance (6.48% and 4.10%) in predicting strategy choices (see model 1a and model 1b in Table 4). Although this finding does not support the findings of Heinemann et al. [16] and Schmidt et al. [17], it does agree with that of Büyükboyacı [12], who found that an individual’s propensity to choose the uncertain action depended on the others’ risk attitudes but not on the decision-maker’s own risk attitudes. This finding also aligns with Al-Ubaydli et al. [23], who found no correlation, and partly with that of Guarin and Babin [22], who found only a small correlation between a player’s risk attitude and their actions in a stag hunt game. Nevertheless, risk attitudes could have an indirect effect on strategy choices through their effect on the FOBs and SOBs, as our results suggest. The effect size of this relationship, however, was small.

Our analysis of the task order effect has shown that the difference in marginal effects of probabilistic FOB on strategy choices was significant in only one out of six pairwise comparisons: the FOB had a greater effect on strategy choices of participants who completed the tasks in the order *SOB*, *FOB*, *strategy selection*, *lottery choices* (second task order treatment) than on the strategy choices of those who completed the tasks in the order *strategy selection*, *SOB*, *FOB*, *lottery choices* (fourth task order treatment). One possible interpretation of this finding is that reporting beliefs before making strategy choices (i.e., fourth treatment) leads participants to rely more heavily on their beliefs when they choose a strategy. However, this finding and interpretation should be treated with caution, as all other comparisons, including those involving SOB, were not significant. From this viewpoint, our study provides only weak evidence for the existence of a task order effect in a stag hunt game. This conclusion is in agreement with the findings of Manski and Neri [21] and of Costa-Gomes and Weizsäcker [38], both of who used different game designs and found that the order of the belief elicitation task and the strategy choice task did not play a role in participants’ decisions.

### 5.1. Implications

Applying our findings to the example of coordinating protests that we described in the introduction, these may imply that whether or not a potential protester will join a protest depends on their expectation about whether coordination will be achieved (e.g., a sufficiently high number of people will participate), as well as on whether they believe that the others expect them to join a protest. In contrast, whether the person is risk-averse, neutral, or seeking is less important in determining their willingness to participate in protest activities. The implication for the second example given in the introduction (in which mask-wearing is treated as a coordination problem) could be that measures influencing individuals’ normative expectations (i.e., SOBs) can be more effective in inducing a welfare-maximizing behavior (e.g., everybody wears a mask) than those appealing to the empirical expectation (i.e., FOBs). That means, if one wants to manipulate the behavior of the decision-maker in both examples, it seems more effective to affect the normative expectations of this person (e.g., through information that influences expectations in relation to the expectations of others with regard to one’s own behavior (cf. Berninghaus et al. 2013)). In other words, one must be able to manipulate the decision-maker’s own expectation regarding the image that others have of them in the desired direction.

Importantly, the inferences made in the preceding paragraphs are based on the assumption that our findings are generalizable to real-world settings and coordination situations beyond the specific one that our study investigated. Nevertheless, when considering studies on protest participation, we note that our findings are in accordance with those of Born et al. [52]. In their case study of a cleaners’ strike, the authors found the likelihood that the workers’ willingness to engage in the same strike behavior (to strike vs. not to strike) increases when they trust one another. Although beliefs are not exactly the same as trust, the two terms share expectations about others’ behavior [53,54]. Against this background, a certain degree of comparison is possible between our results and Born et al.’s [52]. Similar to their findings, participants in our experiment imitated the behavior they expected from others. In a broader sense, there is also an overlap between the findings of research on mask-wearing behavior and those of this study. In their work, Nakayachi et al. [55] investigated factors that could explain why people choose to wear masks. They found that societal norms were the main factor driving the decision to wear a mask, while expectations regarding the risk of infection played only a minor role.

### 5.2. Limitations and Directions for Future Research

The present study is subject to several limitations. First, we only examined one type of coordination game, the stag hunt game, and it remains unclear whether our results can be generalized to other types of coordination games. It is also uncertain whether our results would differ if we used a different exchange rate to convert points into euros (e.g., 10 points = €6 instead of 100 points = €6). It is possible, for example, that risk attitudes could have a greater effect on strategy choices when higher payoffs were involved. Hence, further research is needed using other types of coordination games and different exchange rates. Second, the sample comprised university students from Germany, which may limit the generalizability of the findings to other populations. However, the meta-analysis by Zumaeta [56] found no statistically significant differences between student and non-student samples in coordination behavior, even though the findings of Cooper [57] suggest otherwise. The third limitation relates to the belief-elicitation mechanism (i.e., QSR) used in this study to measure probabilistic FOBs and SOBs. According to Danz et al. [58], QSR can lead to center-biased reporting of beliefs. Future research using alternative belief-elicitation methods is needed to ensure the robustness of our results (see Charness et al. [31] for an overview of belief-elicitation methods). 

## 6. Conclusions

To conclude, this study contributes to the existing literature on using experimental methods to investigate the decisions individuals make in games with multiple equilibria. Our findings reveal that understanding individuals’ beliefs is key to understanding how humans make choices in these types of games. Both FOBs and SOBs were found to be strong predictors of coordination behavior, with a slightly stronger predictive power observed for SOBs. This may imply that measures influencing individual normative expectations (i.e., second-order beliefs or SOBs) can be more effective in inducing welfare-maximizing behavior than those appealing to the empirical expectation (i.e., first-order beliefs or FOBs). However, further research is needed to determine the conditions under which these results hold true. From a methodological point of view, we have contributed to the question of whether the order in which participants complete tasks affects the relationship between the two belief types (FOB and SOB) and particular strategy choices.

## Figures and Tables

**Table 1:** Lottery choices.

Lottery Pair No.	Lottery A	Lottery B
	[1 − p, 200; p, 0]	[p, 150; 1 − p, 120]
1	[0.99, 200; 0.01, 0]	[0.01, 150; 0.99, 120]
2	[0.95, 200; 0.05, 0]	[0.05, 150; 0.95, 120]
3	[0.90, 200; 0.10, 0]	[0.10, 150; 0.90, 120]
4	[0.80, 200; 0.20, 0]	[0.20, 150; 0.80, 120]
5	[0.70, 200; 0.30, 0]	[0.30, 150; 0.70, 120]
6	[0.60, 200; 0.40, 0]	[0.40, 150; 0.60, 120]
7	[0.50, 200; 0.50, 0]	[0.50, 150; 0.50, 120]
8	[0.40, 200; 0.60, 0]	[0.60, 150; 0.40, 120]
9	[0.30, 200; 0.70, 0]	[0.70, 150; 0.30, 120]
10	[0.20, 200; 0.80, 0]	[0.80, 150; 0.20, 120]
11	[0.10, 200; 0.90, 0]	[0.90, 150; 0.10, 120]
12	[0.05, 200; 0.95, 0]	[0.95, 150; 0.05, 120]
13	[0.01, 200; 0.99, 0]	[0.99, 150; 0.01, 120]

**Table 2:** Range of possible payoffs and task order within the experimental conditions.

	Strategy Selection	FOB Elicitation	SOB Elicitation	Lottery Choices
Possible payoffs (100 Points = €6)				
Minimum payoff	€0.00	€0.00	€0.00	€0.00
Riskless payoff	€7.20	€4.50	€4.50	€7.20
Maximum payoff	€12.00	€6.00	€6.00	€12.00
Task order 1	1	2	3	4
Task order 2	1	3	2	4
Task order 3	3	1	2	4
Task order 4	3	2	1	4

**Table 3:** Strategy selection, qualitative FOB, and qualitative SOB in the coordination game.

Game Behavior	Number of Players
Strategy selection	
Strategy A (payoff dominant)	118 (83.10%)
Strategy B (risk dominant)	24 (16.90%)
Qualitative FOB	
Strategy A	119 (83.80%)
Strategy B	23 (16.20%)
Qualitative SOB	
Strategy A	123 (86.62%)
Strategy B	19 (13.38%)
N=	142

Note. Descriptive data showing participants’ strategy choices and their qualitative FOB and SOB. Qualitative FOB = participants’ qualitative beliefs regarding the partner’s strategy choice; Qualitative SOB = participants’ qualitative beliefs regarding the partner’s qualitative beliefs about which strategy they had chosen.

**Table 4:** Results of the logit regression analyses predicting strategy selections.

	Model 1a			Model 1b		
	B (S.E.)	OR	Wald (*p*-Value)	B (S.E.)	OR	Wald (*p*-Value)
prob. FOB	−7.894 (1.704)	0.00004	21.44 (<0.001)	-		-
prob. SOB	-		-	−11.110 (2.566)	0.00002	18.70 (<0.001)
Risk attitude	2.011 (1.978)	7.477	1.04 (0.309)	2.829 (2.378)	16.921	1.41 (0.234)
Constant	1.653 (1.928)	5.222	0.86 (0.391)	2.690 (2.390)	14.724	1.28 (0.261)
N	142			142		
LR χ^2^ (2)	49.19 (*p* < 0.001)			65.18 (*p* < 0.001)		
McFadden’s *R*^2^	0.381			0.505		
Log-L.	−39.92			−31.92		

Note. Dependent variable is the strategy selection (0 = Strategy A, 1 = Strategy B). prob. FOB = participants’ probabilistic first-order belief that the partner had chosen Strategy A, prob. SOB = participants’ probabilistic second-order belief that the partner believes they themselves had chosen Strategy A, LR = Likelihood Ratio, Log-L. = Log-Likelihood.

**Table 5:** Results of the linear regression analyses predicting probabilistic FOB and SOB.

	Model 2a (DV = pr. FOB)		Model 2b (DV = pr. SOB)	
	B (S.E.)	*t* (*p*-Value)	B (S.E.)	*t* (*p*-Value)
Risk attitude	−0.351 (0.143)	−2.45 (0.016)	−0.357 (0.144)	−2.48 (0.014)
Constant	1.034 (0.115)	8.98 (<0.001)	1.038 (0.115)	9.01 (<0.001)
N	142		142	
F(1, 140)	6.00 (*p* < 0.001)		6.17 (*p* < 0.001)	
*R* ^2^	0.041		0.042	

Note. prob. FOB = participants’ probabilistic first-order belief that the partner had chosen Strategy A, prob. SOB = participants’ probabilistic second-order belief that the partner believes they themselves had chosen Strategy A.

## Data Availability

The data presented in this study are available in the Supplementary Materials.

## Supplementary Materials

The following supporting information can be downloaded at: https://www.mdpi.com/article/10.3390/bs13030228/s1, Supplementary File S1: Experimental data.

## Appendix A

### Appendix A.1. Experimental Instruction for the Strategy Selection Task

(Task 1, page 1)

Instruction

On the next page, you will be presented with a two-person game. In this game, you and a randomly selected player (“other player”) will be faced with a choice between two strategies: Strategy A and Strategy B. As can be seen in the *game matrix*, the outcome of each strategy depends not only on your own strategy choice but also on that of the other player, resulting in four possible strategy combinations (A,A), (A,B), (B,A), and (B,B). The first number in a field represents your payoff, and the second number represents the payoff of the other player.

In this part of the survey, we would like you to indicate which strategy you prefer.

(Task 1, page 2)

Decision (Replica of the computer-based response screen.)

Game matrix				Other player	
				Strategy A	Strategy B
		You	Strategy A	200/200	0/120
			Strategy B	120/0	150/150
				Strategy A	Strategy B
Which strategy do you prefer?				**□**	**□**

### Appendix A.2. Experimental Instruction for the First-Order Belief Elicitation Task

(Task 2, page 1)

Instruction

On the next page, you will be presented with a two-person game. In this game, you and a randomly selected player (“other player”) will be faced with a choice between two strategies: Strategy A and Strategy B. As can be seen in the *game matrix*, the outcome of each strategy depends not only on your own strategy choice but also on that of the other player, resulting in four possible strategy combinations (A,A), (A,B), (B,A), and (B,B). The first number in a field represents your payoff, and the second number represents the payoff of the other player.

In this part of the survey questionnaire, we would like you to:

(1) indicate your guess about what strategy the other player will choose and;
(2) assign a number *p* between 0 (absolutely uncertain) and 100 (absolutely certain) that best describes how certain you are about your guess.

Payoff mechanism

Your payoff will be determined as follows:

**Case 1:** If your guess about the other player’s strategic choice is correct, your payoff will be determined by the following formula:		Example for the possible payoffs, if the guess is correct, as a function of the different values of *p*:	
$100\cdot \left[1-{\left(1-\frac{p}{100}\right)}^{2}\right]$	p = degree of certainty	p = 0 → 0 Points	p = 60 → 84 Points
		p = 10 → 19 Points	p = 70 → 91 Points
		p = 20 → 36 Points	p = 80 → 96 Points
		p = 30 → 51 Points	p = 90 → 99 Points
		p = 40 → 64 Points	p = 100 → 100 Points
		p = 50 → 75 Points	
**Case 2:** If your guess about the other player’s strategic choice is wrong, your payoff will be determined by the following formula:		Example for the possible payoffs if the guess is wrong, as a function of the different values of p:	
$100\cdot \left[1-{\left(\frac{p}{100}\right)}^{2}\right]$	p = degree of certainty	p = 0 → 100 Points	p = 60 → 64 Points
		p = 10 → 99 Points	p = 70 → 51 Points
		p = 20 → 96 Points	p = 80 → 36 Points
		p = 30 → 91 Points	p = 90 → 19 Points
		p = 40 → 84 Points	p = 100 → 0 Points
		p = 50 → 75 Points	

(Task 2, page 2)

Decision (Replica of the computer-based response screen.)

Please indicate which strategy you think the other player will choose and indicate how confident (0–100) you are of your guess.

Game matrix				Other player	
				Strategy A	Strategy B
		You	Strategy A	200/200	0/120
			Strategy B	120/0	150/150
				Strategy A	Strategy B
Which strategy do you think the other player will choose?				□	□
How confident (0–100) are you of your guess? Please enter a number between 0 (absolutely uncertain) and 100 (absolutely certain).					
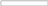

### Appendix A.3. Experimental Instruction for the second-Order Belief Elicitation Task

(Task 3, page 1)

Instruction

On the next page, you will be presented with a two-person game. In this game, you and a randomly selected player (“other player”) will be faced with a choice between two strategies: Strategy A and Strategy B. As can be seen in the *game matrix*, the outcome of each strategy depends not only on your own strategy choice but also on that of the other player, resulting in four possible strategy combinations (A,A), (A,B), (B,A), and (B,B). The first number in a field represents your payoff, and the second number represents the payoff of the other player.

In this part of the survey questionnaire, we would like you to:

(1) indicate your guess about what the other player believes about what strategy you will choose and;
(2) assign a number *p* between 0 (absolutely uncertain) and 100 (absolutely certain) that best describes how certain you are about your guess.

Payoff mechanism

Your payoff will be determined as follows:

**Case 1:** If your guess about the other player’s strategic choice is correct, your payoff will be determined by the following formula:		Example for the possible payoffs, if the guess is correct, as a function of the different values of *p*:	
$100\cdot \left[1-{\left(1-\frac{p}{100}\right)}^{2}\right]$	p = degree of certainty	p = 0 → 0 Points	p = 60 → 84 Points
		p = 10 → 19 Points	p = 70 → 91 Points
		p = 20 → 36 Points	p = 80 → 96 Points
		p = 30 → 51 Points	p = 90 → 99 Points
		p = 40 → 64 Points	p = 100 → 100 Points
		p = 50 → 75 Points	
**Case 2:** If your guess about the other player’s strategic choice is wrong, your payoff will be determined by the following formula:		Example for the possible payoffs if the guess is wrong, as a function of the different values of p:	
$100\cdot \left[1-{\left(\frac{p}{100}\right)}^{2}\right]$	p = degree of certainty	p = 0 → 100 Points	p = 60 → 64 Points
		p = 10 → 99 Points	p = 70 → 51 Points
		p = 20 → 96 Points	p = 80 → 36 Points
		p = 30 → 91 Points	p = 90 → 19 Points
		p = 40 → 84 Points	p = 100 → 0 Points
		p = 50 → 75 Points	

(Task 3, page 2)

Decision (Replica of the computer-based response screen.)

Please indicate which strategy you think the other player will choose and indicate how confident (0–100) you are of your guess.

Game matrix				Other player	
				Strategy A	Strategy B
		You	Strategy A	200/200	0/120
			Strategy B	120/0	150/150
				Strategy A	Strategy B
What do you think the other player believes you would choose?				□	□
How confident (0–100) are you of your guess? Please enter a number between 0 (absolutely uncertain) and 100 (absolutely certain).					

### Appendix A.4. Experimental Instruction for the Lottery Choice Task

(Task 4, page 1)

Instruction

In this part of the survey, you will be presented with 13 lottery pairs. For each of the lottery pairs, please indicate which of the two lotteries (A or B) you prefer. The lotteries are all of the following type:

	**Lottery A**		**Lottery B**	
probability	p%	(100-p)%	p%	(100-p)%
payoff	G_1A_	G_2A_	G_1B_	G_2A_

For each lottery, p denotes the probability to earn the amount of points G_1A_ or G_1B_ and (100-p) denotes the probability to win the amount of money G_2A_ or G_2B_.

Example

	**Lottery A**		**Lottery B**	
probability	10%	90%	90%	10%
payoff	300	100	500	0

In this example: if you choose lottery A you will earn 300 points with a probability of 10% and 100 points with a probability of 90%. If you choose lottery B you will earn 500 points with a probability of 90% and 0 points with a probability of 10%.

Payoff mechanism

At the end of the experiment, one of the decisions will be realized. This decision will be determined by drawing one ball from a bingo cage containing nine balls numbered from 1 to 13. According to your preferences, the preferred lottery will be played by drawing a ball from a bingo cage containing a specified number of red and blue balls, reflecting the probabilities of the lottery (the number of red balls equates to the probability of the payoff one (G_1_) and the number of blue balls equates to the probability of the payoff two (G_2_)). In the case of indifference, the toss of a coin determines which lottery is played (heads for lottery A, tails for lottery B).

(Task 4, page 2)

Decision

Please use the response form on the next page to indicate your decision for each of the 13 lottery pairs, whether you prefer lottery A or B. If you are indifferent between the two lotteries, please check the box “indifferent”.
